# PEPNet: a two-stage point cloud framework with hierarchical embedding and antigen–antibody interaction modeling for epitope prediction

**DOI:** 10.1093/bib/bbag067

**Published:** 2026-02-19

**Authors:** Jiayi Chen, Guixu Zhang, Zhijian Xu, Qian Zhang

**Affiliations:** School of Computer Science and Technology, East China Normal University, 3663 Zhongshan North Road, Putuo District, Shanghai 200062, China; School of Computer Science and Technology, East China Normal University, 3663 Zhongshan North Road, Putuo District, Shanghai 200062, China; Shanghai Frontiers Science Center of Optogenetic Techniques for Cell Metabolism, Shanghai Key Laboratory of New Drug Design, State Key Laboratory of Bioreactor Engineering, School of Pharmacy, East China University of Science and Technology, 130 Meilong Road, Xuhui District, Shanghai 200237, China; State Key Laboratory of Drug Research, Shanghai Institute of Materia Medica and Drug Discovery and Design Center, Shanghai Institute of Materia Medica, Chinese Academy of Sciences, 555 Zuchongzhi Road, Pudong New District, Shanghai 201203, China; School of Computer Science and Technology, East China Normal University, 3663 Zhongshan North Road, Putuo District, Shanghai 200062, China; Shanghai Frontiers Science Center of Molecule Intelligent Syntheses, East China Normal University, 3663 Zhongshan North Road, Putuo District, Shanghai 200062, China

**Keywords:** 3D point cloud, epitope prediction, pretraining strategy, antigen–antibody interactions, hierarchical embedding

## Abstract

Epitope prediction is a key challenge in immunology and therapeutic antibody design. Existing computational methods rely on residue-level graph representations that fail to capture fine-grained atomic-level geometric information essential for antibody–antigen recognition. Considering that protein structure files (e.g. Protein Data Bank (PDB) files) inherently contain 3D atomic coordinates, we model proteins as atomic-level point clouds to directly preserve high-resolution spatial features . Building on this representation, we propose Point cloud-based Epitope Prediction Network(PEPNet), a two-stage point cloud framework for epitope prediction. Inspired by the natural atom-to-residue hierarchy in proteins, PEPNet employs a residue-aware hierarchical embedding module to aggregate atomic features into residue-level embeddings. To capture sequential dependencies absent in unordered point clouds, we integrate rotary positional encoding. Additionally, PEPNet leverages a BERT-style pretraining strategy with data augmentation to mitigate data scarcity, and a cross-attention decoder to explicitly model antigen–antibody interactions. Experimental results show that PEPNet achieves the best overall performance (MCC = 0.401, AUC = 0.765). Even when evaluated on AlphaFold3-predicted structures, PEPNet maintains strong robustness (MCC = 0.346), still outperforming WALLE (MCC = 0.305). These results underscore PEPNet’s potential for real-world antibody–antigen analysis and design.

## Introduction

Epitopes are specific surface residues on antigens that are recognized and bound by antibodies, thereby initiating immune responses [1, 2]. B-cell epitopes (BCEs) are regions on the antigen surface that can be recognized by B-cell receptors and subsequently by secreted antibodies, playing a key role in humoral immunity [3, 4]. Notably, $\sim$90% of BCEs are conformational, comprising discontinuous amino acid residues that are brought into proximity through protein folding, making their identification particularly challenging [5, 6]. This emphasizes the importance of accurately representing the 3D spatial organization of antigens, as subtle geometric and atomic-level features are essential for capturing the nature of antibody recognition. Accurate epitope prediction is critical for subunit vaccine design [7, 8], therapeutic antibody development [9], and disease diagnosis [10].

Traditional experimental methods are costly, complex, and have low throughput [11–13], making computational models a highly promising alternative. Computational approaches for BCE prediction have evolved through three paradigms. (i) **Sequence-based** methods predict epitopes directly from the primary amino-acid sequence, without using 3D structures. They have increasingly benefited from protein language models (PLMs) [14, 15], which provide context-aware embeddings that improve over handcrafted sequence descriptors. Early representatives such as BepiPred-2.0 [16] and CBTOPE [16] rely on handcrafted physicochemical features, whereas more recent work incorporates PLM embeddings or fine-tunes PLMs on residue-level labels—e.g. ESMBind [17] fine-tunes ESM-2 with LoRA [18] on UniProt binding-site annotations. And ESMFold [14] represents a breakthrough in structure prediction by training a folding head on ESM-2, enabling end-to-end protein structure prediction from sequence alone without requiring multiple sequence alignment and thereby allowing epitope inference directly from predicted structures. While sequence-based methods are simple and broadly applicable—especially when structural information is unavailable—they inherently struggle to capture protein structural information that is known to be the determinants of protein functions. (ii) **Structure-based** methods leverage conformational information to address this limitation. EpiPred [19] adopts a graph-based scoring function to evaluate geometric complementarity between antigens and antibodies. MaSIF-site [20] applies geometric deep learning to protein surfaces (represented as meshes with encoded geometric and physicochemical descriptors) to predict binding sites. Nevertheless, they often fail to make full use of the complementary information across different modalities within limited data. (iii) To overcome the limitations of single-modality inputs, **multimodal fusion** models have emerged, aiming to jointly leverage features from both antigen (structure and/or sequence) and antibody (structure and/or sequence). WALLE [21] represents graph nodes with PLM embeddings, thereby fusing sequence-derived features with structural topology.

**Figure 1:**
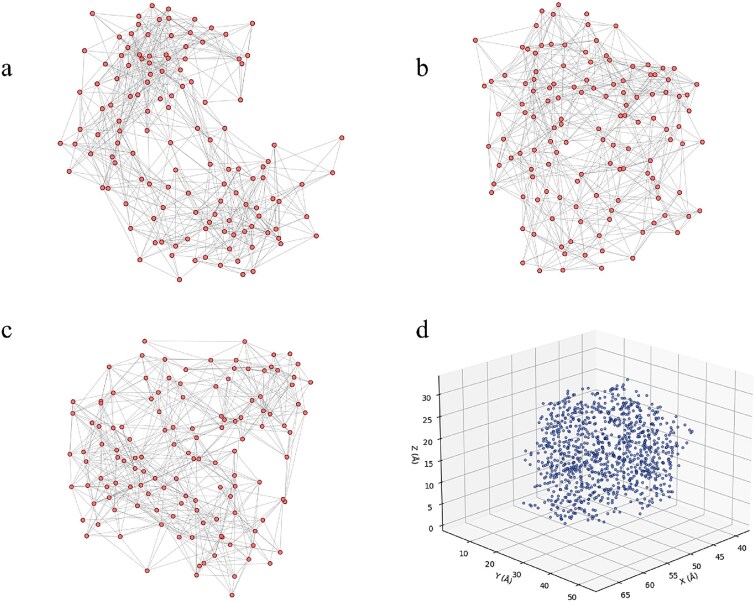
Comparison of graph-based and point cloud representations for protein structure modeling using the antigen chain from AbDb 2dqd_0P. (a–c) Graph-based representations, where each node represents a residue, and an edge $e_{ij}=1$ is established if any non-hydrogen atom of residues $i$ and $j$ are within 4.5 Å. The three subfigures correspond to visualizations with different random seeds for the layout algorithm, showing that the node arrangement in 2D space varies arbitrarily and does not reflect the actual 3D geometry of the protein. (d) Atom-level point cloud representation of the same structure, where each point corresponds to the atom coordinate, directly preserving the true spatial geometry of the protein.

Despite great advances in geometric deep learning, structural representations in epitope prediction primarily employ conventional residue-level graphs [21–24]. Such graphs typically rely on predefined distance thresholds to construct edges that discretize the continuous 3D space. This process may discard fine-grained geometric information, including directional and angular dependencies, that are critical for modeling antigen–antibody interactions. Inspired by recent advances in 3D point clouds in the computer vision domain, we propose to view proteins as atomic-level 3D point clouds, where each atom serves as a spatial point carrying rich physicochemical attributes. This paradigm naturally preserves high-resolution geometric continuity and offers a principled way to model spatial hierarchies within protein structures (see Fig. 1). However, directly transferring 3D point cloud frameworks from vision tasks to molecular systems introduces domain mismatches.

Common local-aggregation strategies such as Farthest-Point Sampling (FPS) with k-Nearest Neighbors (KNN) or voxelization partition space in a task-agnostic way and therefore ignore the intrinsic atom-residue hierarchy of proteins [25–27]. In addition, existing approaches treat atoms as the basic units and simply append residue-level descriptors to atomic features (i.e. direct feature concatenation) [28], which creates redundant representations and fails to learn meaningful cross-level fusion between atomic and residue information. Another challenge arises from the unordered nature of point clouds that conflicts with the sequential order inherently present in proteins. Finally, available Ag–Ab datasets remain relatively small (e.g. MIPE: 626 pairs; Episcan: 162 complexes; EpiPred: 148 complexes) [19, 29, 30], which further constrains data-hungry deep learning models.

In this work, we present PEPNet (**P**oint cloud-based **E**pitope **P**rediction **Net**work), a novel framework that addresses the limitations of residue-level graph representations by modeling proteins as atomic-level 3D point clouds. Building on this atomic-level representation, we introduce several key innovations. First, inspired by the natural atom-to-residue hierarchy in protein structures, we propose a residue-aware hierarchical embedding module (RHEM) that explicitly leverages this biological organization to aggregate atomic features into residue-level embeddings, effectively bridging the atomic and residue scales. Second, to address the inherent orderlessness of point clouds—which conflicts with the sequential nature of protein chains—we integrate rotary positional encoding(RoPE) to capture chain order information [31]. Third, to mitigate the challenge of limited epitope training data, we design a BERT-style self-supervised pretraining strategy where physicochemical properties are masked and reconstructed [32], combined with diverse data augmentation strategies to enhance robustness against structural noise. In the fine-tuning stage, a cross-attention decoder explicitly models antigen–antibody binding interactions, yielding antibody-aware epitope predictions. Extensive experiments demonstrate that PEPNet achieves the best overall performance (MCC = 0.401, AUC = 0.765), and maintains strong robustness even on AlphaFold3-predicted [33] structures (MCC = 0.346), making it a practical and reliable tool for real-world antibody–antigen analysis.

## Materials and methods

### Problem definition

We formulate the epitope prediction task as a binary classification problem defined on antigen residues, given the atomic-level 3D structures of both the antigen and its interacting antibody. Specifically, the model takes the following inputs:

- The atomic coordinates of the antigen and antibody, denoted as $X_{A} \in \mathbb{R}^{N_{A} \times 3}$ and $X_{B} \in \mathbb{R}^{N_{B} \times 3}$, where $N_{A}$ and $N_{B}$ are the number of atoms in the antigen and antibody, respectively;
- The corresponding atomic features, $F_{A}^{\textrm{atom}} \in \mathbb{R}^{N_{A} \times d_{a}}$ and $F_{B}^{\textrm{atom}} \in \mathbb{R}^{N_{B} \times d_{a}}$;
- The residue-level features, such as physicochemical properties and optional embeddings from pretrained PLMs, denoted as $F_{A}^{\textrm{res}} \in \mathbb{R}^{R_{A} \times d_{r}}$ and $F_{B}^{\textrm{res}} \in \mathbb{R}^{R_{B} \times d_{r}}$, where $R_{A}$ and $R_{B}$ are the number of residues in the antigen and antibody, respectively;
- A predefined atom-to-residue mapping, $\mathcal{M}_{A}: \{1, \dots , N_{A}\} \rightarrow \{1, \dots , R_{A}\}$ and $\mathcal{M}_{B}: \{1, \dots , N_{B}\} \rightarrow \{1, \dots , R_{B}\}$, which associate each atom with a residue index. In practice, this can be represented by a list of start indices marking the first atom of each residue.

The goal is to predict, for each antigen residue, a probability $\hat{y}_{i} \in [0, 1]$ indicating whether it is part of a conformational BCE. The ground truth is given as a binary label vector $y \in \{0, 1\}^{R_{A}}$, where $y_{i} = 1$ denotes that residue $i$ is an epitope residue. A residue is labeled as an epitope if any of its heavy atoms is within 4.5 Å of any heavy atom of the antibody.

### Data preparation

We chose AsEP [21] for our experiments, as it is currently the largest publicly available benchmark for epitope prediction that provides antibody–antigen complex structures. AsEP consists of 1723 non-redundant antibody–antigen complexes with annotated residue-level epitope labels, curated from AbDb [34], and processed into graph-based representations.

We strictly follow the official train/validation/test splits released by AsEP, without performing any additional re-splitting. We additionally report antigen sequence and structure similarity distributions between the training and test sets for these splits in Appendix 1.

*Epitope-Ratio Split.* Complexes are split such that the ratio of epitope residues to antigen surface residues is approximately consistent across training, validation, and test sets. This stratified sampling ensures that the classification difficulty remains comparable across all subsets. The final split contains 1383 complexes for training, and 170 each for validation and testing.

*Epitope-Group Split.* Complexes are grouped by epitope groups. The dataset is split to ensure that test-set epitope groups are disjoint from those in the training and validation sets, enabling rigorous evaluation of generalization to unseen binding regions. We use the same 80%/10%/10% train/val/test split ratio.

### Protein representations

AsEP only provides the graph-format data and does not include physicochemical properties of proteins. Since our model operates directly on the 3D atomic point cloud, we do not use the official graph format of AsEP. Instead, we reconstruct a compatible point-cloud dataset from the raw structural files provided by the authors and recompute both atomic-level and residue-level features.

Specifically, we utilize the structures.tar.gz archive, which contains 1723 antibody–antigen complex structures in PDB format [35]. For each complex, we preprocess the raw files as follows: (i) remove all solvent molecules, metal ions, and ligands using PyMOL [36]; (ii) repair missing atoms within existing residues using PDBFixer [37]; (iii) compute atomic-level features; and (iv) assign residue-level features. An overview of the extracted input features is summarized in Table 1 (see details in Appendix 2).

**Table 1:** Description of input features used in the PEPNet, grouped by their conceptual category: AF (atomic-level features), RF (residue-level physicochemical features), and LE (PLM embedding)

**Type**	**Feature type**	**Dim**
**AF**	3D coordinates	3
	Atom type (C, N, O, S, H)	5
	Local surface normal vector	3
**RF**	Amino acid type (e.g. ALA, ARG, $\ldots$)	20
	Position-specific scoring matrix (PSSM) obtained using PSI-BLAST [38]	20
	Solvent accessibility	2
	Neighbor composition	20
**LE**	Protein language model embedding	480 (Ag)/512 (Ab)

### Model architecture

**Figure 2:**
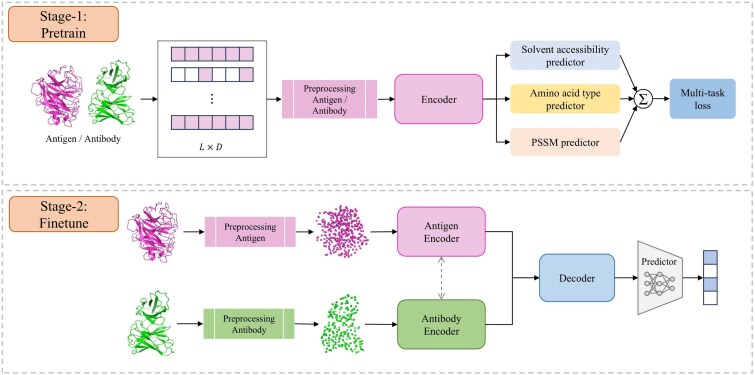
Overview of the proposed PEPNet framework. In Stage 1, the model is pretrained by masking and reconstructing residue-level physicochemical attributes in a self-supervised manner. For implementation convenience, the input of Stage 1 is the antigen–antibody complex, which is split into individual chains and processed separately without interaction. In Stage 2, a cross-attention decoder captures antigen–antibody interactions to perform residue-level epitope classification.

To address the challenge of accurate epitope prediction, we design PEPNet, a two-stage framework that integrates pretraining and fine-tuning. In the pretraining stage, PEPNet learns generalizable structural and physicochemical representations by masking residue-level attributes and reconstructing them in a self-supervised manner. In the fine-tuning stage, a decoder is introduced to model antigen–antibody interactions via cross-attention, enabling residue-level classification of epitopes. This hierarchical design allows PEPNet to first acquire a broad understanding of the intrinsic relationships within proteins and then specialize in antigen–antibody binding prediction. An overview of the model architecture is provided in Fig. 2.

#### Data augmentation

Despite AsEP representing the largest manually curated antibody–antigen benchmark dataset to date, the training set comprises only 1383 antigen–antibody pairs. Furthermore, physicochemical attributes calculated using various tools are often subject to noise. To mitigate the limitations imposed by both the scarcity of data and the presence of noise, we implemented several data augmentation strategies. These strategies include: (i) introducing Gaussian noise to atomic normal vectors and coordinates; (ii) applying random rotations to the entire antigen and antibody structures independently; and (iii) adding noise to amino acid features, such as neighbor composition and solvent accessibility, during the pretraining phase.

#### PEPNet-pretrain

##### Residue-aware hierarchical embedding module

The RHEM is a critical preprocessing step in PEPNet, designed to hierarchically encode atomic and residue-level information. This module bridges the gap between fine-grained atomic features and residue-level representations.

**Figure 3:**
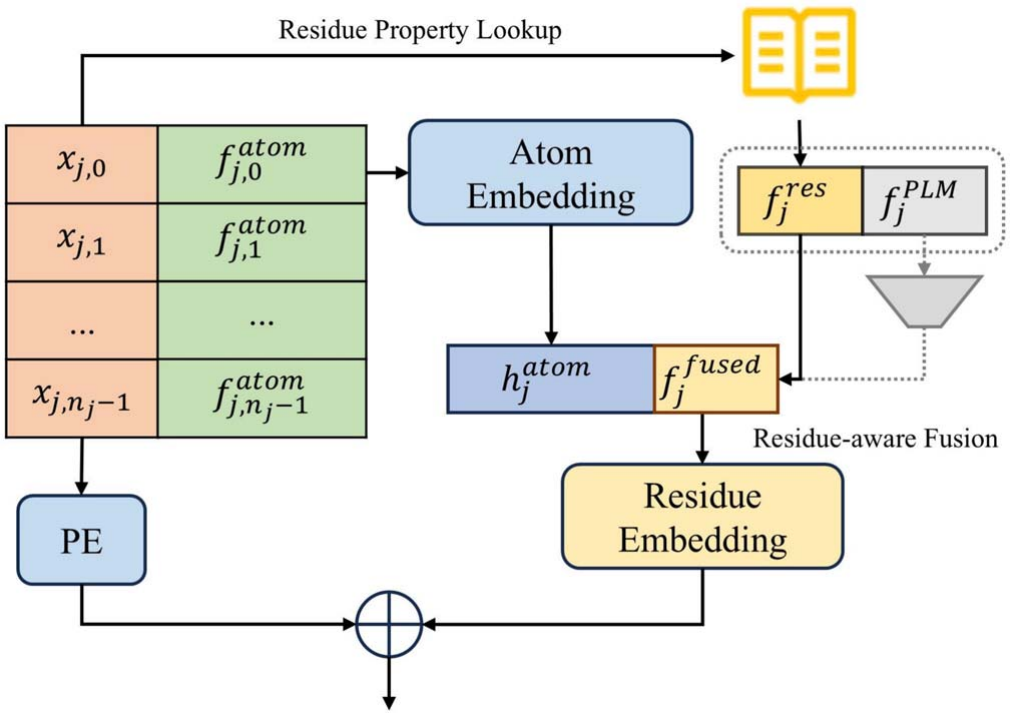
The RHEM in PEPNet, serving as a preprocessing stage. The gray part indicates the processing of optional PLM embeddings.

As illustrated in Fig. 3, each amino acid residue $r_{j}$ is composed of $n_{j}$ atoms. For each atom $k$ within residue $r_{j}$ (where $0 \leq k < n_{j}$), we consider its 3D coordinates $x_{j,k} \in \mathbb{R}^{3}$ and other 8D atomic-level descriptors $f^{\textrm{atom}}_{j,k} \in \mathbb{R}^{8}$. These two components are concatenated to form an initial 11D feature vector $[x_{j,k} \| f^{\textrm{atom}}_{j,k}]$ for each atom. These atomic features are embedded and aggregated into a residue-level atomic representation $h^{\textrm{atom}}_{j}$ through a lightweight PointNet module [25, 39], which primarily consists of MLPs and max pooling layers, over all atoms in residue $r_{j}$.

In parallel, we extract residue-level physicochemical features $f^{\textrm{res}}_{j} \in \mathbb{R}^{62}$ via a residue property lookup table.

Optionally, we incorporate contextual residue embeddings from PLMs to enhance feature expressiveness. For each residue $r_{j}$, we denote the extracted PLM embedding as $f^{\textrm{PLM}}_{j} \in \mathbb{R}^{d_{\textrm{PLM}}}$.

We first concatenate the residue-level physicochemical features and PLM embeddings, then apply a multi-layer perceptron (MLP) for joint dimensionality reduction followed by dropout regularization:


1 \begin{eqnarray*}& f_{j}^{\textrm{fused}} = \textrm{Dropout}(\textrm{MLP}([f^{\textrm{res}}_{j} \| f^{\textrm{PLM}}_{j}]))\end{eqnarray*}

where the MLP consists of two linear layers with GELU activation.

We then fuse the atomic and processed residue-level features by concatenation and feed the resulting vector into a residue embedding module. To further encode positional information of residues within the chain, a learnable positional encoding (PE) is added. The final residue representation is defined as:


2 \begin{eqnarray*}& h_{j} = \textrm{Embed}([h^{\textrm{atom}}_{j} \| f_{j}^{\textrm{fused}}]) + \textrm{PE}_{j}\end{eqnarray*}

where ∥ denotes concatenation. The function $\textrm{Embed}(\cdot )$ is also implemented as a lightweight PointNet module (see details in Appendix 3). If PLM embeddings are not available, we simply set $f_{j}^{\textrm{fused}} = f^{\textrm{res}}_{j}$. These residue embeddings serve as input to the encoder in the following stage.

##### Encoder

**Figure 4:**
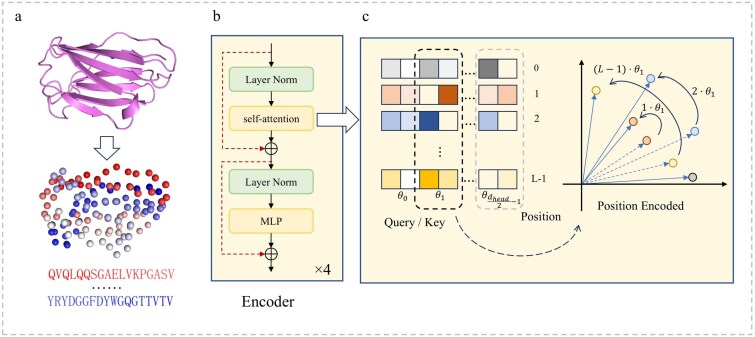
Illustration of the encoder architecture. (a) Protein structures are transformed into residue-level point clouds. (b) Each encoder block consists of LN, multi-head self-attention, and feed-forward layers with residual connections. (c) RoPE rotates query and key vectors by position-dependent angles, so that their inner products encode relative positional distances between residues.

For both antigen and antibody, we employ a shared encoder to extract residue-level representations. The use of shared weights effectively doubles the training data available for each parameter, thereby facilitating more sufficient learning and reducing the risk of overfitting. Formally, let $H^{\textrm{A}} \in \mathbb{R}^{R_{A} \times d}$ and $H^{\textrm{B}} \in \mathbb{R}^{R_{B} \times d}$ denote the antigen and antibody residue-level representations, respectively. The overall structure of the encoder is illustrated in Fig. 4b. The encoder is composed of four stacked Transformer blocks. Each block follows a pre-norm design and comprises: a Layer Normalization (LN), a multi-head self-attention layer followed by a residual connection, and then another LN and an MLP followed by a second residual connection.

In addition to the absolute PE derived from atomic coordinates, we argue that the sequential order of residues plays a crucial role in defining the biological semantics of protein chains. Unlike natural scene point clouds that are inherently unordered, protein sequences are strictly ordered, and permuting residues alters their biological interpretation (Fig. 4a). To address this, we introduce RoPE that implicitly incorporates sequence order into the self-attention mechanism by rotating the query and key vectors (Fig. 4c).

Given $H$, the encoder first projects it into query, key, and value spaces via learned linear mapping:


3 \begin{eqnarray*}& Q = H W_{q}, \quad K = H W_{k}, \quad V = H W_{v}\end{eqnarray*}

We employ multi-head self-attention with $l$ heads. Specifically, the projected features are reshaped into $l$ subspaces:


4 \begin{eqnarray*}& Q \rightarrow \{Q^{(t)}\}_{t=1}^{l}, \quad K \rightarrow \{K^{(t)}\}_{t=1}^{l}, \quad V \rightarrow \{V^{(t)}\}_{t=1}^{l}\end{eqnarray*}

where each head has dimension $d_{\textrm{head}} = d / l$. To incorporate sequential information, we apply RoPE to the query and key vectors of each head by rotating every 2D subspace. Concretely, for a 2D slice $(q^{(t)}_{2i}, q^{(t)}_{2i+1})$ of the query vector $q^{(t)}_{m}$ at position $m$, the rotation is defined as:


5 \begin{eqnarray*}& \begin{bmatrix} q^{\prime (t)}_{2i} \\ q^{\prime (t)}_{2i+1} \end{bmatrix} = \begin{bmatrix} \cos(m\theta_{i}) & -\sin(m\theta_{i}) \\ \sin(m\theta_{i}) & \cos(m\theta_{i}) \end{bmatrix} \begin{bmatrix} q^{(t)}_{2i} \\ q^{(t)}_{2i+1} \end{bmatrix}\end{eqnarray*}

where $\theta _{i}$ is a frequency term controlling the rotation speed in the $i$th subspace. Following the standard RoPE formulation, the frequencies are defined as


6 \begin{eqnarray*}& \theta_{i} = \textrm{base}^{-2i/d_{\textrm{head}}}, \quad i = 0, 1, \dots, \frac{d_{\textrm{head}}}{2}-1\end{eqnarray*}

where $d_{\textrm{head}}$ denotes the rotary embedding dimension and *base* controls the spectrum of frequencies. While the original RoPE commonly uses $\mathit{base}=10\,000$, we set $\mathit{base}=2048$ in PEPNet to better match the sequence-length characteristics of antigen and antibody chains and to achieve a balanced decay behavior of attention. A detailed analysis of the effect of different base values is provided in the Appendix 4. The same transformation is applied to the key vector $k^{(t)}_{n}$ at position $n$. When the rotated query $q^{\prime (t)}_{m}$ and key $k^{\prime (t)}_{n}$ are multiplied, their inner product implicitly encodes the relative distance $(n-m)$, thereby introducing sequence-order information into the attention mechanism.

The self-attention layer for head $t$ is computed as:


7 \begin{eqnarray*}& \textrm{Attention}^{(t)}(Q^{\prime (t)}, K^{\prime (t)}, V^{(t)}) = \textrm{softmax}\!\left( \frac{Q^{\prime (t)} {K^{\prime (t)}}^{\top}}{\sqrt{d_{\textrm{head}}}} \right)V^{(t)}\end{eqnarray*}

The outputs of all heads are then concatenated and linearly projected to form the final multi-head attention output.

##### Pretraining objective

Inspired by the self-supervised learning paradigm of BERT, we adopt a BERT-style pretraining strategy for protein feature modeling. Specifically, we focus on three types of residue-level physicochemical features: amino acid type, solvent accessibility, and PSSM.

**Figure 5:**
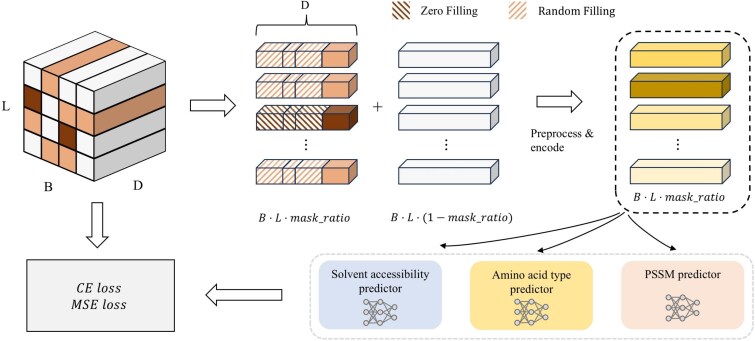
Overview of the pretraining objective in PEPNet-pretrain. In each mini-batch, 50% of residues on the antigen and antibody chains are randomly selected for masking. The masked features are replaced by zero filling (20%) or random filling (80%) and combined with unmasked features before being processed by the encoder. The model reconstructs only the masked features through three task-specific prediction heads: amino acid-type classification, solvent accessibility regression, and PSSM regression. The training objective is a weighted sum of cross-entropy and mean squared error losses.

In each mini-batch, we randomly mask 50% of the residues independently on the antigen and antibody chains. The masked features are then replaced with zero vectors (20% probability) or with randomly sampled values within the feature-specific range (80% probability). The corrupted features are concatenated with the unmasked features and subsequently processed by the encoder for representation learning (Fig. 5).

We enforce feature reconstruction only on the masked residues. To this end, we employ three parallel prediction heads: (i) a 20-class classifier for amino acid type prediction, (ii) a 2D regressor for solvent accessibility prediction, and (iii) a 20D regressor for PSSM reconstruction. Each prediction head shares the same architecture: an MLP consisting of two linear layers with GELU activation and a dropout layer. The prediction head can be formulated as:


8 \begin{eqnarray*}& p = \textrm{Linear}(\textrm{Dropout}(\textrm{GELU}(\textrm{Linear}(h^{\prime}))))\end{eqnarray*}

The overall pretraining loss is a weighted combination of cross-entropy and mean squared error (MSE):


9 \begin{eqnarray*}& L = \lambda_{\textrm{aa}} L_{\textrm{aa}} + \lambda_{\textrm{sol}} L_{\textrm{sol}} + \lambda_{\textrm{pssm}} L_{\textrm{pssm}}\end{eqnarray*}

where $L_{\textrm{aa}}$ is the cross-entropy loss for amino acid type prediction; $L_{\textrm{sol}}$ and $L_{\textrm{pssm}}$ are MSE losses for solvent accessibility and PSSM reconstruction, respectively.

#### PEPNet-finetune

The overall architecture of PEPNet-finetune closely follows the pretrained model, with RHEM and encoder weights directly initialized from PEPNet-pretrain. This design ensures that the encoder can leverage residue-level representations enriched by self-supervised learning.

#### Decoder

To capture antigen–antibody binding interactions, we introduce a cross-interaction decoder that enables residue-level information flow from the antibody to the antigen. Unlike a standard Transformer decoder, we remove self-attention for simplicity.

Formally, let $\widetilde{H}^{\textrm{A}} \in \mathbb{R}^{R_{A} \times d^{\prime}}$ and $\widetilde{H}^{\textrm{B}} \in \mathbb{R}^{R_{B} \times d^{\prime}}$ denote the antigen and antibody representations, respectively. We initialize $Z^{\textrm{A}} = \widetilde{H}^{\textrm{A}}$, and compute the updated antigen representation as:


10 \begin{eqnarray*} Z^{\prime \textrm{A}} &= Z^{\textrm{A}} + \textrm{Dropout}(\textrm{Attn}(\textrm{LN}({Z}^{\textrm{A}}), \textrm{LN}(\widetilde{H}^{\textrm{B}}), \textrm{LN}(\widetilde{H}^{\textrm{B}}))) \end{eqnarray*}

11 \begin{eqnarray*} \widetilde{Z}^{\textrm{A}} &= Z^{\prime \textrm{A}} + \textrm{Dropout}(\textrm{MLP}(\textrm{LN}(Z^{\prime \textrm{A}}))) \end{eqnarray*}

Here, $\textrm{Attn}(Q, K, V)$ denotes the multi-head cross-attention operation with query $Q$, key $K$, and value $V$, respectively, while $\textrm{LN}$ represents layer normalization. The antibody embeddings $\widetilde{H}^{\textrm{B}}$ serve as both keys and values, ensuring that each antigen residue explicitly incorporates antibody-derived context.

The resulting output $\widetilde{Z}^{\textrm{A}}$ is thus an antibody-aware antigen embedding, which is subsequently passed to the prediction head for residue-level epitope classification.

#### Finetuning objective

For epitope prediction, the embedding of each residue on the antigen, denoted as $\widetilde{z}^{a}$, derived from the decoder is inputted into an MLP-based predictor, $F_{\textrm{site}}$. This predictor assesses the probability of a residue being the epitope:


12 \begin{eqnarray*}& \hat{y}= F_{\textrm{site}}(\widetilde{z}^{a})\end{eqnarray*}

Specifically, $F_{\textrm{site}}$ consists of two fully connected layers with hidden size 256, each followed by LN, ReLU activation, and Dropout. These are followed by a final linear projection layer and a sigmoid activation to produce the epitope probability.

The model is trained using a simple Binary Cross-Entropy Loss.

The Binary Cross-Entropy Loss is given by:


13 \begin{eqnarray*}& L_{\textrm{BCE}} = -\frac{1}{N} \sum_{i=1}^{N} \left[ y_{i} \log(\hat{y}_{i}) + (1 - y_{i}) \log(1 - \hat{y}_{i}) \right]\end{eqnarray*}

where $y_{i} \in \{0, 1\}$ is the ground truth label for residue $i$, $\hat{y}_{i}$ is the predicted probability, and $N$ is the total number of antigen residues. Label smoothing is also applied to soften the hard labels and improve generalization.

### Performance evaluation

Given the binary nature and class imbalance of the task (epitopes vs. non-epitopes), we adopt multiple evaluation metrics to provide a comprehensive assessment.

We highlight the Matthews correlation coefficient (**MCC**) as the primary evaluation metric, following recent recommendations [21, 40], due to its ability to account for all four outcomes in the confusion matrix and its robustness under class imbalance. For completeness and comparability with prior work, we also report Area Under the ROC Curve (AUC), Precision, Recall, and F1-score. Notably, all threshold-dependent metrics (MCC, Precision, Recall, and F1-score) are calculated using a probability threshold optimized on the validation set to maximize the MCC.

### Implementation

We implemented PEPNet using PyTorch and trained the model in two stages, i.e. pretraining and fine-tuning. In both stages, we employed the AdamW optimizer with a learning rate of 1e-4 and a weight decay of 0.05. A cosine learning rate schedule was applied, and warm-up epochs were used to stabilize early training.

The pretraining stage was conducted for 300 epochs, with the first 10 epochs used for linear warm-up. We set the total batch size to 96. During fine-tuning, the model was further optimized for 400 epochs. A longer warm-up of 100 epochs was used to adapt the pretrained parameters to the downstream prediction task. The total batch size was set to 64. All experiments were performed on a single Linux machine (Ubuntu 18.04.6) with two NVIDIA RTX3090 GPU cards.

## Experimental results

### The overall performance of PEPNet

To comprehensively evaluate the performance of our method on the epitope prediction task, we compared PEPNet with five representative baselines, including two sequence-based methods (ESMFold and ESMBind), two structure-based methods (EpiPred and MaSIF-site), and one multimodal method (WALLE). All models were assessed on the AsEP dataset under two evaluation protocols: splitting by the epitope-to-antigen surface ratio (Ratio) and by epitope groups (Group). The comparative results are summarized in Table 2.

**Table 2:** Performance on dataset split by epitope to antigen surface ratio (Ratio) and by epitope groups (Group)

				**Ratio**					**Group**	
	**MCC**	**AUC**	**Precision**	**Recall**	**F1**	**MCC**	**AUC**	**Precision**	**Recall**	**F1**
*Sequence-based methods*										
ESMFold [14]	0.028	–	0.137	0.043	0.060	0.018	–	0.113	0.034	0.046
ESMBind [17]	0.016	0.506	0.106	0.121	0.090	0.002	0.500	0.082	0.076	0.064
*Structure-based methods*										
EpiPred [19]	0.029	–	0.122	0.180	0.142	−0.006	–	0.089	0.158	0.112
MaSIF-site [20]	0.037	–	0.125	0.183	0.114	0.046	–	0.164	0.174	0.128
PEPNet	**0.401**	**0.765**	**0.544**	0.340	**0.419**	0.139	0.612	0.230	0.143	0.177
PEPNet(AlphaFold3)	0.346	0.751	0.497	0.286	0.363	0.119	0.601	0.208	0.125	0.156
*Multimodal methods*										
WALLE [21]	0.305	0.695	0.308	**0.516**	0.357	0.152	0.596	0.207	**0.299**	**0.204**
PEPNet+LE	0.337	**0.765**	0.465	0.295	0.361	**0.156**	**0.627**	**0.250**	0.155	0.191
PEPNet+LE(AlphaFold3)	0.332	0.758	0.471	0.282	0.353	0.145	0.621	0.239	0.145	0.180

The highest score in each column is in bold.

On the Ratio split, PEPNet consistently outperforms all competing methods across most metrics. In particular, PEPNet achieves the best MCC (0.401), AUC (0.765), Precision (0.544), and F1 (0.419). Specifically, the MCC of PEPNet surpasses the second-best WALLE (0.305) by 31.5%, while its AUC improves by 10.1% (0.765 vs. 0.695). The advantage is even more pronounced in terms of Precision, where PEPNet achieves a 76.6% relative improvement (0.544 vs. 0.308). Although its Recall (0.340) is lower than that of WALLE (0.516), the balanced performance across other metrics highlights the overall superiority of PEPNet. Since MCC is known to be robust under class imbalance and AUC provides a threshold-independent measure of overall predictive ability, these results clearly demonstrate the effectiveness of PEPNet in this setting.

We further evaluated the robustness of PEPNet when provided with predicted structures from AlphaFold3 [33], denoted as PEPNet(AlphaFold3). While the performance declines compared with experimental structures (MCC: 0.346 vs. 0.401; AUC: 0.751 vs. 0.765), it remains substantially better than all other baselines. For example, compared with WALLE, PEPNet(AlphaFold3) improves MCC by 13.4% and AUC by 8.1%. This demonstrates that PEPNet maintains strong generalization ability even when applied to predicted rather than experimentally resolved structures.

On the Group split, which poses a more challenging scenario by requiring prediction on previously unseen epitope groups, PEPNet+LE achieves the best performance in terms of MCC, AUC, and Precision. Specifically, it attains an MCC of 0.156, representing a 2.6% improvement over the second-best WALLE (0.152). Its AUC reaches 0.627, surpassing WALLE (0.596) by 5.2%, while its Precision improves to 0.250, a 20.8% gain over WALLE (0.207). Although the F1 score of PEPNet+LE (0.191) is slightly lower than that of WALLE (0.204), the better performance in MCC and AUC highlights its stronger overall predictive capability. Moreover, PEPNet+LE demonstrates robust performance when applied to AlphaFold3-predicted structures: compared with experimental structures, the MCC decreases by only 0.011 and the AUC by just 0.006, underscoring the stability and generalizability of the proposed framework. In addition, we provide the two-stage training-validation curves of the proposed models (see details in Appendix 5).

Interestingly, we observe divergent effects of PLM embeddings under different data partitioning strategies. In the ratio split, where training and test sets share antigens with similar sequence patterns, the addition of PLM features introduces redundancy with physicochemical and structural descriptors, occasionally amplifying noise and reducing overall performance. In contrast, the group split poses a more challenging generalization scenario, as the epitope groups in the test set are not observed during training. Here, the evolutionary and long-range dependencies captured by PLMs complement structural information and provide transferable representations across antigens, leading to consistent performance gains.

To align with prior work [21], we include MaSIF-site as a representative antibody-agnostic baseline. For completeness, we further evaluated and analyzed comparisons with SEMA-1D and SEMA-3D [41] (see details in Appendix 6.1).

### The effectiveness of pretraining

**Figure 6:**
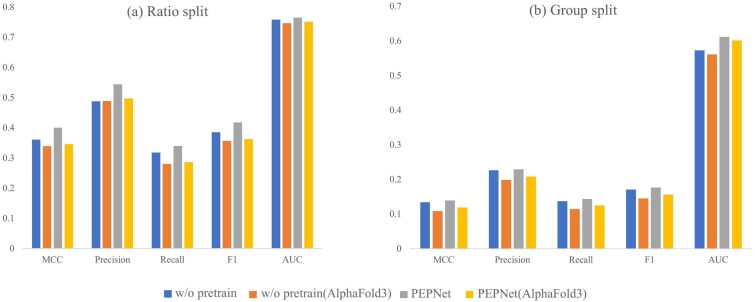
Ablation study on the effect of pretraining under the ratio and group splits.

To assess the contribution of the two-stage training paradigm, we conducted an ablation study comparing PEPNet with and without pretraining under both ratio and group splits (Fig. 6 and Appendix Table S4). Overall, pretraining consistently enhances model performance across most evaluation metrics. Under the ratio split, PEPNet with pretraining achieves an MCC of 0.401 and an AUC of 0.765, representing relative improvements of 10.8% and 0.8% over the non-pretrained counterpart, respectively. And under the group split, the pretrained model also yields similar gains, with an AUC improvement from 0.573 to 0.612 and an MCC increase from 0.134 to 0.139.

### The effectiveness of position encoding

To clarify the respective roles of absolute PE and RoPE, we conducted ablation experiments comparing PE only (w/o RoPE), RoPE only (w/o PE), and their combination (PEPNet) under both ratio and group splits (Table 3).

**Table 3:** Ablation results of position encoding under ratio and group splits

				**Ratio**					**Group**	
	**MCC**	**AUC**	**Precision**	**Recall**	**F1**	**MCC**	**AUC**	**Precision**	**Recall**	**F1**
w/o RoPE	0.346	0.742	0.529	0.265	0.353	0.092	0.590	0.199	0.082	0.117
w/o RoPE (AlphaFold3)	0.341	0.745	0.535	0.255	0.345	0.097	0.582	0.208	0.084	0.119
w/o PE	0.389	**0.784**	**0.571**	0.304	0.397	0.120	0.548	0.212	0.122	0.155
w/o PE (AlphaFold3)	0.357	0.772	0.545	0.271	0.362	0.122	0.547	0.219	0.120	0.155
PEPNet	**0.401**	0.765	0.544	**0.340**	**0.419**	**0.139**	**0.612**	**0.230**	**0.143**	**0.177**
PEPNet (AlphaFold3)	0.346	0.751	0.497	0.286	0.363	0.119	0.601	0.208	0.125	0.156

The highest score in each column is in bold and the second-best score is underlined.

On experimentally resolved structures, RoPE plays a dominant role in improving performance. Removing RoPE (w/o RoPE) results in substantial degradation across most metrics, whereas the RoPE-only variant generally outperforms the PE-only baseline. Importantly, the full PEPNet model yields the best overall performance. For example, on the ratio split, PEPNet improves MCC from 0.389 to 0.401 and F1 from 0.397 to 0.419, while on the group split, it achieves the highest MCC (0.139) and F1 (0.177). Overall, these results indicate that RoPE is crucial for capturing biologically meaningful residue order within self-attention, while PE provides complementary positional cues at the embedding level.

When evaluated on AlphaFold3-predicted structures, we observe that the performance gains brought by RoPE become less consistent compared with those on experimentally resolved structures. One possible explanation is the discrepancy between ATMSEQ, which is derived from experimentally resolved coordinates and used during training, and SEQRES, which represents the full-length sequence in AlphaFold3 inputs. This mismatch may introduce mild sequence-structure misalignments, potentially weakening the effectiveness of RoPE’s sequence-order encoding.

In future work, such effects could be mitigated through several complementary strategies. First, sequence-aligned indexing could be adopted, in which RoPE positional indices are assigned according to the absolute residue positions in SEQRES rather than the compressed order of ATMSEQ, thus preserving biologically meaningful sequence distances. Second, missing-segment simulation could be incorporated as a form of data augmentation during training; e.g. a small fraction of residues could be randomly deleted. Third, incorporating predicted structures, such as those generated by AlphaFold3, into the training pipeline would provide complete coordinate representations and more consistent positional signals.

### The effectiveness of residue-aware hierarchical embedding module

To assess the effectiveness of the proposed RHEM, we compared the full PEPNet-scratch model with a simplified variant in which RHEM is replaced by direct spatial grouping based on FPS and KNN (Table 4). For simplicity, all models in this experiment are trained from scratch without the two-stage learning scheme.

**Table 4:** Ablation results of RHEM under ratio and group splits

				**Ratio**					**Group**	
	**MCC**	**AUC**	**Precision**	**Recall**	**F1**	**MCC**	**AUC**	**Precision**	**Recall**	**F1**
w/o RHEM	0.312	0.738	0.357	**0.355**	0.356	0.100	**0.606**	0.146	**0.179**	0.161
w/o RHEM (AlphaFold3)	0.287	0.747	0.344	0.319	0.331	0.079	0.600	0.128	0.159	0.142
PEPNet-scratch	**0.362**	**0.759**	0.488	0.318	**0.385**	**0.134**	0.573	**0.227**	0.137	**0.171**
PEPNet-scratch (AlphaFold3)	0.339	0.747	**0.489**	0.281	0.357	0.109	0.561	0.198	0.114	0.145

The highest score in each column is highlighted in bold.

Under the ratio split, the model equipped with RHEM achieves an MCC of 0.362 and an F1 of 0.385, outperforming the simplified version (MCC 0.312, F1 0.356). A similar trend is observed in the group split, where the model with RHEM attains an MCC of 0.134 compared with 0.100 for the version without it. These results highlight that RHEM effectively strengthens residue-level representations by leveraging the natural atom-residue hierarchy, thereby providing biologically meaningful patches. Overall, this ablation confirms that the performance gains of PEPNet stem in part from RHEM’s ability to incorporate domain-specific prior knowledge into the feature aggregation process, rather than relying solely on local spatial adjacency.

We further analyze the effects of data augmentation and feature types (see details in Appendices 6.2 and 6.3). Overall, the ablation results indicate that data augmentation plays a critical role in generalization, particularly for AlphaFold3-predicted structures. Without augmentation, models exhibit inflated performance on experimental data but degrade substantially on predicted structures, whereas the full augmentation pipeline yields more stable performance across settings. In addition, the feature-type ablation study reveals complementary strengths between traditional physicochemical features and PLM embeddings.

### Case study

To further evaluate the interpretability and biological relevance of the proposed model, we conducted case studies on two representative antigen–antibody complexes selected from the ratio and group splits. To examine whether these cases overlap with the training set, we evaluated both sequence and structural similarity. Sequence similarity was assessed using MMseqs2 [42] easy-search with default parameters on the antigen sequences, which returned no significant hits for either antigen. Structural similarity was evaluated using TM-score [43], yielding maximum values of 0.31 and 0.22, respectively.

Case 1: Ratio split—6k65_0P.

The first case study is based on the antigen–antibody complex 6k65_0P [44], evaluated under the ratio split and inferred using PEPNet. On this case, the model achieves an MCC of 0.489, an F1 score of 0.583, and an AUC of 0.773, with a Precision of 0.70 and a Recall of 0.50.

Case 2: Group split—4f2m_1P.

The second case study focuses on the complex 4f2m_1P [45], evaluated under the group split and inferred using PEPNet+LE. The model achieves an MCC of 0.571, an F1 score of 0.60, and an AUC of 0.873, with a Precision of 0.50 and a Recall of 0.75.

**Figure 7:**
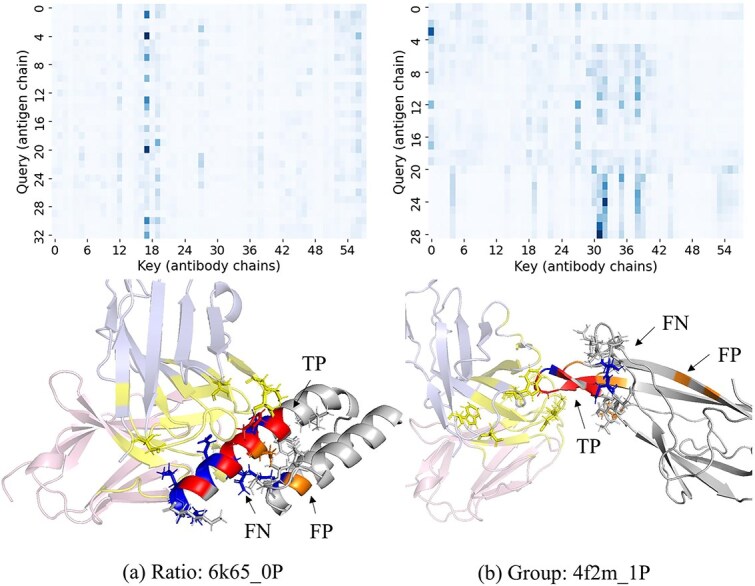
Case study of PEPNet and PEPNet+LE predictions on representative antigen–antibody complexes. (a) 6k65_0P and (b) 4f2m_1P. Top: filtered cross-attention maps between interface residues (y = antigen residues; x = antibody residues). Interface residues are defined as those containing any atom within 8 Å (including hydrogens) of the opposite chain. Bottom: PyMOL structural views of the corresponding antigen–antibody complexes with predicted true positives, false negatives, and false positives indicated. Antibody residues located within 8 Å of the antigen are additionally highlighted. For each complex, the top 20 antigen–antibody residue pairs ranked by attention score are shown in stick representation.

As shown in Fig. 7, several consistent observations can be made across the two cases. First, antigen residues receiving high attention scores largely overlap with experimentally annotated epitope residues. Second, although some residues are incorrectly classified, these residues still exhibit relatively high attention scores, indicating that the model attends to them during the cross-attention process but ultimately fails to assign the correct class label. This observation reveals a potential discrepancy between attention allocation and the final classification decision. Third, antibody residues with high attention scores tend to be concentrated in loop regions.

Further, we conducted a four-stage t-SNE visualization on them, where embeddings were extracted from the atom embedding module, the residue embedding module, the encoder, and the decoder. The details are provided in Appendix Figs S7 and S8. The atom-level embeddings exhibited limited separability, which was slightly improved at the residue level. The encoder further enhanced the cluster structure, while the decoder introduced antibody information, enabling antibody-specific epitope prediction.

## Conclusions

In this paper, motivated by the direct availability of 3D atomic coordinates in protein structure files, which naturally suit point cloud processing, we present PEPNet, a two-stage point cloud framework for epitope prediction. To address the limitations of scarce training data, we introduce a masked reconstruction task on physicochemical properties together with diverse data augmentation strategies, thereby enhancing robustness. Furthermore, we incorporate sequence order into point cloud representations and designed the RHEM to achieve effective domain adaptation for proteins. Comprehensive experiments demonstrated the effectiveness of PEPNet and its components, achieving competitive performance on both ratio and group splits, even when using AlphaFold3-predicted structures. Nevertheless, certain limitations remain. In particular, the performance under the group split is still suboptimal that we attribute to the scarcity and imbalance of available antigen–antibody data. In future work, we aim to address this limitation by developing large-scale pretraining models tailored to protein point clouds, leveraging not only experimentally resolved complexes but also high-quality structures predicted by AlphaFold2 [46]. We hope that this work will inspire broader applications of geometric deep learning techniques in understanding and predicting biomolecular interactions.

Key Points

- We propose Point cloud-based Epitope Prediction Network (PEPNet), the first two-stage point cloud epitope prediction framework.
- We design a residue-aware hierarchical embedding module to effectively aggregate atomic features into residue-level representations and incorporate protein sequence information through rotary positional encoding.
- We introduce a BERT-style pretraining strategy together with multiple data augmentation to enhance robustness.
- Extensive experiments demonstrate PEPNet achieves competitive performance on epitope prediction, even with AlphaFold3-predicted structures.

## Supplementary Material

bbag067_Supplemental_File

## Data Availability

The source code of PEPNet and related data are publicly available at https://github.com/Zhijian-Xu/PEPNet.
