# Supplementary material for: PEPNet: a two-stage point cloud framework with hierarchical embedding and antigen–antibody interaction modeling for epitope prediction
Source: Brief Bioinform. 2026 Feb 19;27(1):bbag067. doi: 10.1093/bib/bbag067 (PMC12919445; doi:10.1093/bib/bbag067)
Supplement: bbag067_Supplemental_File [file bbag067_supplemental_file.pdf]

Appendix for PEPNet: A Two-Stage Point Cloud Framework with Hierarchical Embedding and Antigen-Antibody Interaction Modeling for Epitope Prediction

1 Train–Test Similarity Analysis under AsEP Splits

In this study, we strictly follow the official AsEP [1] benchmark protocol and use its provided train/test splits (epitope-ratio split and epitope-group split) to ensure fair and reproducible comparisons.

We have now quantified both antigen sequence similarity and structure similarity between the training and test sets under these two splitting protocols. For sequence similarity, we aligned each test-set antigen against all training-set antigens using MMseqs2 [2] (`mmseqs easy-search test.fasta train.fasta result.m8 tmp`). For each test antigen, we recorded the maximum sequence identity to any training antigen and plotted the distribution across the test set. Under the ratio split and epitope-group split, MMseqs2 returned alignments for 151/170 and 131/170 test antigens, respectively (the remaining sequences had no hits under the default MMseqs2 settings). The resulting distributions are reported in Fig. S1 (top panels). For structure similarity, we computed TM-score between each test antigen structure and training antigen structures using TM-score [3] (`TMscore -seq test_i.pdb train_j.pdb`). For each test antigen, we retained the maximum TM-score to any training antigen and plotted the distributions for both splitting protocols. These results are shown in Fig. S1 (bottom panels).

Overall, these analyses provide a direct view of train-test redundancy at both the sequence and structure levels and help contextualize the benchmark evaluation.

We further note that in antibody-conditioned epitope prediction, even the same antigen can present different epitopes to different antibodies [4, 5] (see Fig. S2); thus, high global antigen similarity does not necessarily imply identical interface-level supervision. This point is also illustrated in the AsEP benchmark (see AsEP Supplementary Fig. S3), where multiple antibodies bind distinct regions on the same antigen.

2 Details of Protein Representations

2.1 Atomic-level and residue-level physicochemical features

We extract atomic coordinates and atom types as the fundamental atomic-level representation. To capture geometric information, molecular surfaces are computed using MSMS [6] and further processed to obtain atomic normal vectors.

At the residue level, amino acid types are encoded as 20-dimensional one-hot vectors. Evolutionary information is incorporated via position-specific scoring matrices (PSSMs), generated using PSI-BLAST [7] with three iterations against the SwissProt database and keeping default parameters for other settings. Solvent accessibility is quantified using PyRosetta, providing both absolute

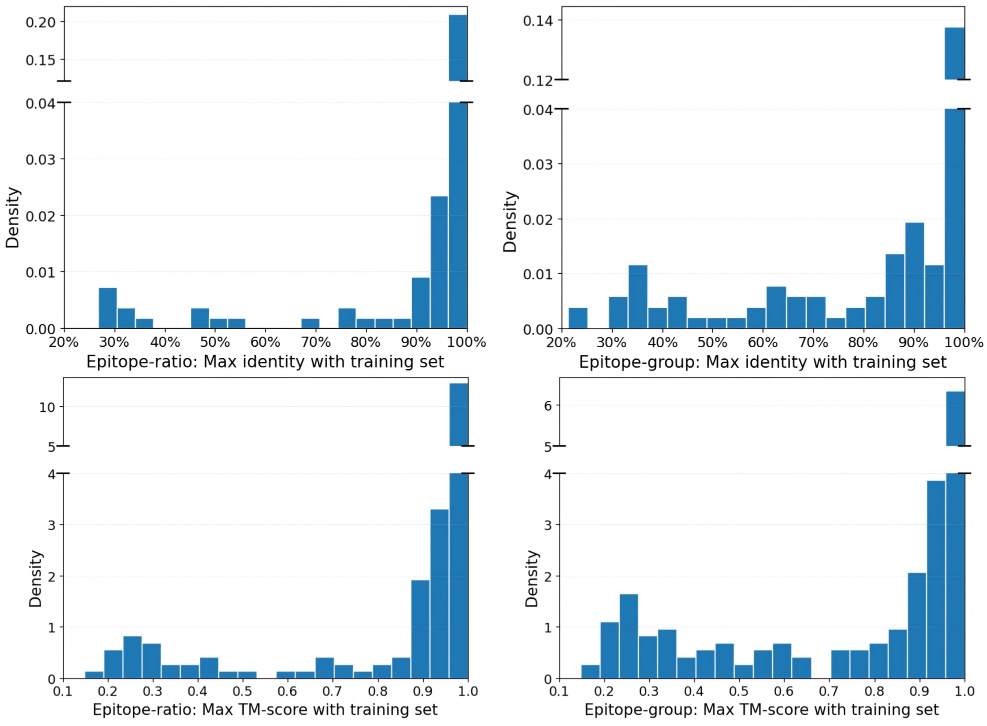

Fig. S1: Distributions of maximum train-test antigen similarity under two splitting protocols.

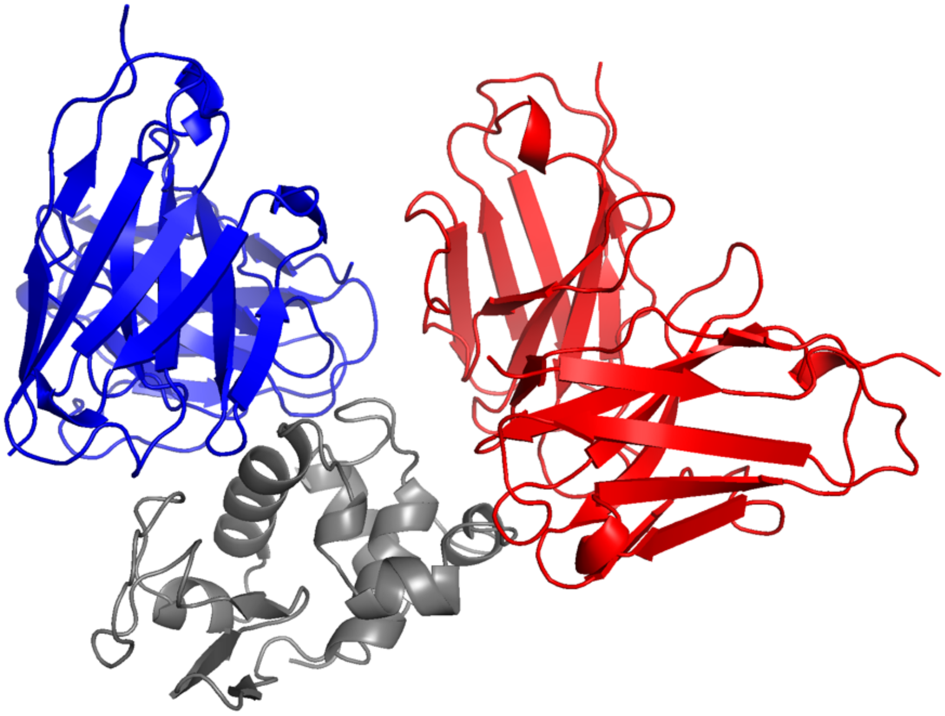

Fig. S2: Structural comparison of two distinct antibody-HEL complexes. The antigen hen egg lysozyme (HEL, gray) is shown bound to two different antibodies: Hy50f (2dqd_0P, blue) and VLW92V (1g7m_0P, red). The structures are superimposed on the HEL antigen to highlight the non-overlapping epitopes recognized by each antibody, illustrating the model's ability to distinguish antibody-specific binding patterns.

and relative solvent-accessible surface area values as a 2-dimensional feature vector for each residue. In addition, we compute neighbor composition: for each residue, the frequencies of the 20 canonical amino acids within an 8 Å neighborhood are calculated.

2.2 Protein language model embedding

In addition to conventional physicochemical features, we incorporate embeddings derived from protein language models (PLMs). PLMs are deep neural networks trained on large corpora of protein sequences, enabling them to capture evolutionary, structural, and functional information in a data-driven manner. For antigen residues, we employ ESM2-35M [8], a transformer-based encoder-only model that follows the BERT [9] paradigm and is trained on the UniRef protein sequence database. For antibody residues, we utilize AntiBERTy [10], a protein language model specifically designed for antibodies. AntiBERTy is trained on 558 million natural antibody sequences using a similar masked-language-modeling objective.

In practice, antibody embeddings are obtained using the official AntiBERTy implementation[1], while antigen embeddings are extracted via the `transformers` library. Given an input sequence of length N, we feed it into the frozen PLM and remove the special start and end tokens. From the final hidden layer, we obtain an $N \times d$ representation matrix, where $d = 512$ for AntiBERTy and $d = 480$ for ESM2. Each row corresponds to the embedding vector of an individual amino acid, providing a contextualized sequence representation that serves as input to our downstream epitope prediction framework.

3 Details of Atom and Residue Embedding Modules

Algorithm 1 Atom Embedding / Residue Embedding Module

Input: Input $\mathbf{X} \in \mathbb{R}^{B \times G \times N \times F}$. B: Batch, G: number of residues in an antigen/antibody.
1: $\mathbf{x} = \text{Reshape}(\mathbf{X}, B \cdot G, N, F)$ # Reshape to combine B and G
2: $\mathbf{x} = \text{ReLU}(\text{LayerNorm}(\text{Conv1d}_{F \to 128}(\mathbf{x})))$ # First block: Conv-LN-ReLU
3: $\mathbf{x}_{\text{local}} = \text{Conv1d}_{128 \to 256}(\mathbf{x})$ # Followed by another Conv
4: $\mathbf{x}_{\text{global}} = \max_N(\mathbf{x}_{\text{local}})$ # Aggregate global features (Max Pool)
5: $\mathbf{x}_{\text{cat}} = \text{Concat}([\mathbf{x}_{\text{local}}, \text{Expand}(\mathbf{x}_{\text{global}}, N)])$ # Fuse global with local features
6: $\mathbf{x} = \text{ReLU}(\text{LayerNorm}(\text{Conv1d}_{512 \to 512}(\mathbf{x}_{\text{cat}})))$ # Second block: Conv-LN-ReLU
7: $\mathbf{x}_{\text{out}} = \text{Conv1d}_{512 \to C}(\mathbf{x})$ # Followed by another Conv
8: $\mathbf{z} = \max_N(\mathbf{x}_{\text{out}})$ # Final max pooling to obtain embeddings
Output: $\mathbf{Z} = \text{Reshape}(\mathbf{z}, B, G, C)$

We provide the pseudocode for the atom and residue embedding modules (see Algorithm 1). Both modules share a similar PointNet-style architecture, and the key difference lies in their input dimensionality. For the atom embedding module, G denotes the number of residues, N denotes the number of atoms per residue, and F denotes the feature dimension of each atom. For the residue embedding module, G also denotes the number of residues, but $N = 1$, and F denotes the residue-level input feature dimension, formed by concatenating the aggregated atomic representation produced by the atom embedding module with residue-level features. In both modules, C denotes the output embedding dimension.

[1] https://github.com/jeffreyruffolo/AntiBERTy

4 Rotary Positional Encoding Frequency Analysis

Frequency formulation in RoPE. In rotary positional encoding (RoPE), positional information is incorporated by rotating query and key vectors in each two-dimensional subspace with position-dependent angles. For the i-th rotary subspace, the rotation frequency is defined as

$$\theta_i = \text{base}^{-2i/d_{\text{head}}}, \quad i = 0, 1, \ldots, \frac{d_{\text{head}}}{2} - 1 \tag{1}$$

where d_{head} denotes the rotary embedding dimension and *base* controls the spectrum of frequencies.

Effect of the base parameter. The choice of *base* directly affects the decay behavior of relative positional similarity induced by RoPE. Following the standard RoPE intuition, we analyze this effect by initializing query and key vectors q and k as all-one vectors. We fix the query position at $m = 0$ and vary the key position n, and compute the inner product

$$\langle q'_m, k'_n \rangle \tag{2}$$

after applying RoPE, as a function of the relative distance $(n - m)$.

As illustrated in Fig. S3, larger *base* values lead to slower decay and lower oscillation frequency of relative positional similarity, making the attention less sensitive to positional differences over shorter distances. In contrast, smaller *base* values result in faster decay and higher oscillation frequency, emphasizing local positional differences. However, excessively small *base* values may suppress the characteristic oscillatory decay pattern, which can be detrimental to stable relative position encoding.

Choice of base in PEPNet. We further examine the sequence-length distribution of antigen and antibody chains in our datasets (Fig. S4), where the maximum sequence length is approximately 1700 residues. Considering this length regime, we select $\textit{base} = 2048$ to provide an appropriate decay behavior of relative positional similarity, allowing the model to capture long-range dependencies while maintaining sufficient sensitivity to positional differences within typical antigen-antibody sequences.

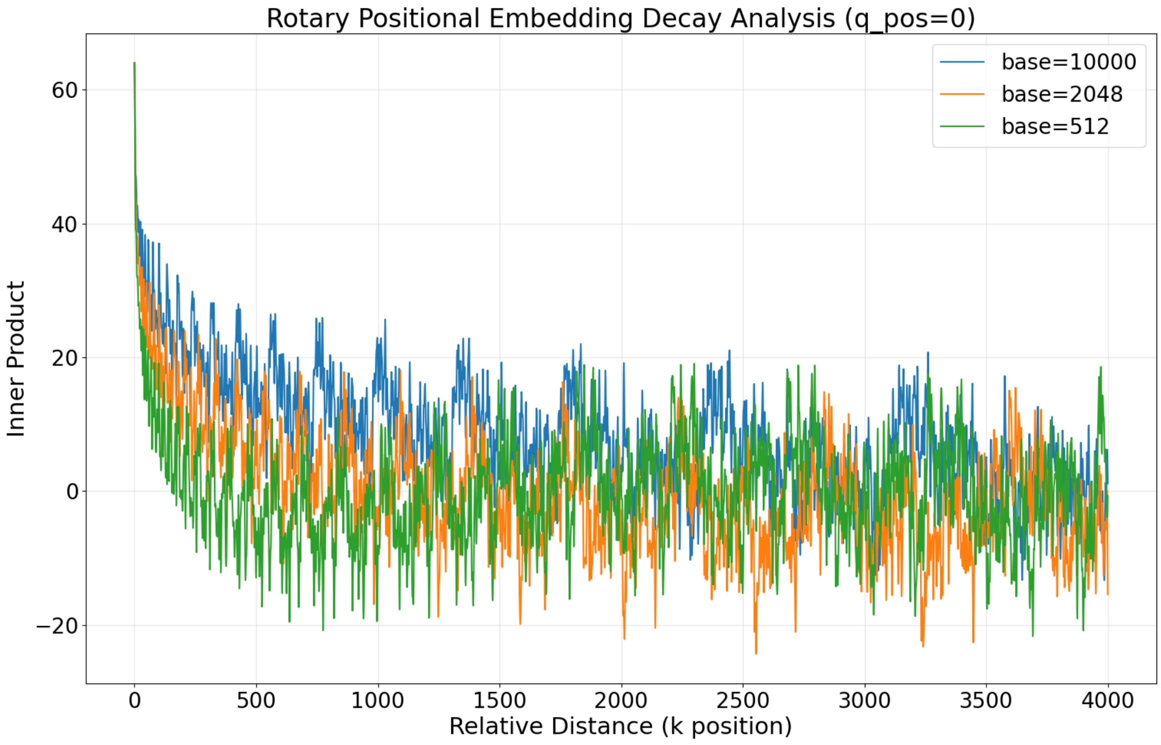

Fig. S3: RoPE decay analysis under different bases.

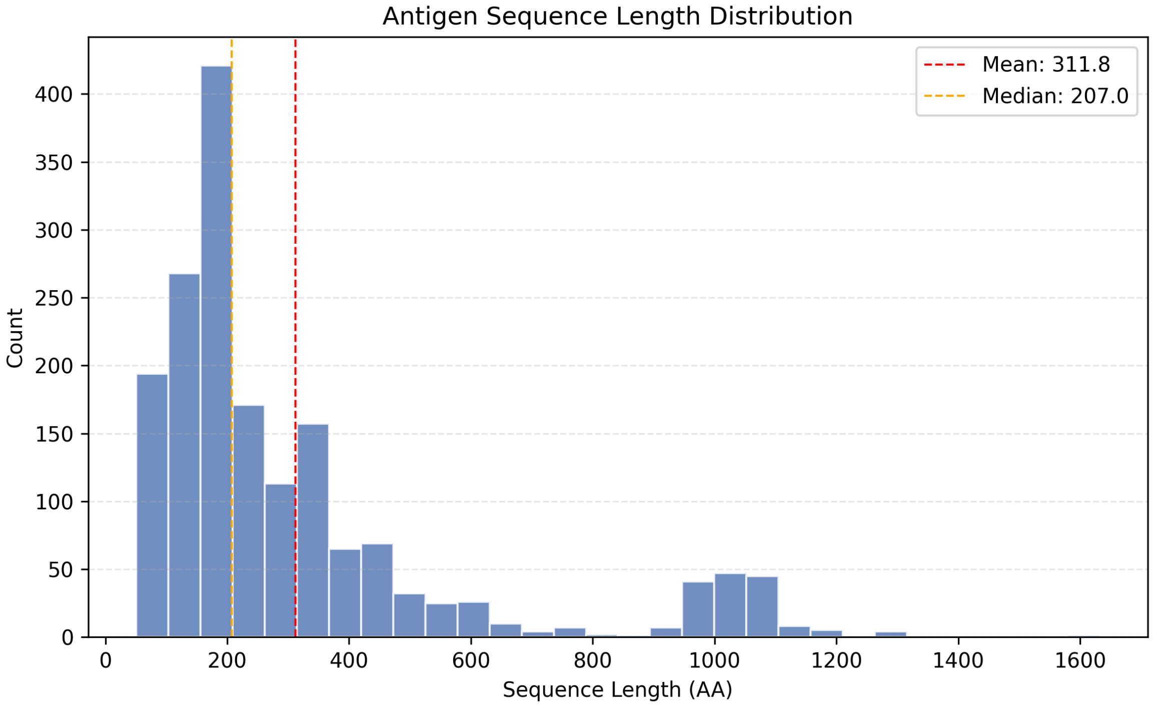

Fig. S4: Antigen sequence length distribution in AsEP.

5 Training-Validation Curves

To assess the training stability and potential overfitting, we report the training-validation curves for both data-splitting strategies used in this study.

For PEPNet under the ratio split, we show the training and validation loss during the pretraining stage, as well as the training loss and validation MCC during fine-tuning (see Fig. S5). For PEPNet+LE under the epitope-group split, the same training protocol and evaluation metrics are applied, and the corresponding curves for both pretraining and fine-tuning stages are reported. (see Fig. S6)

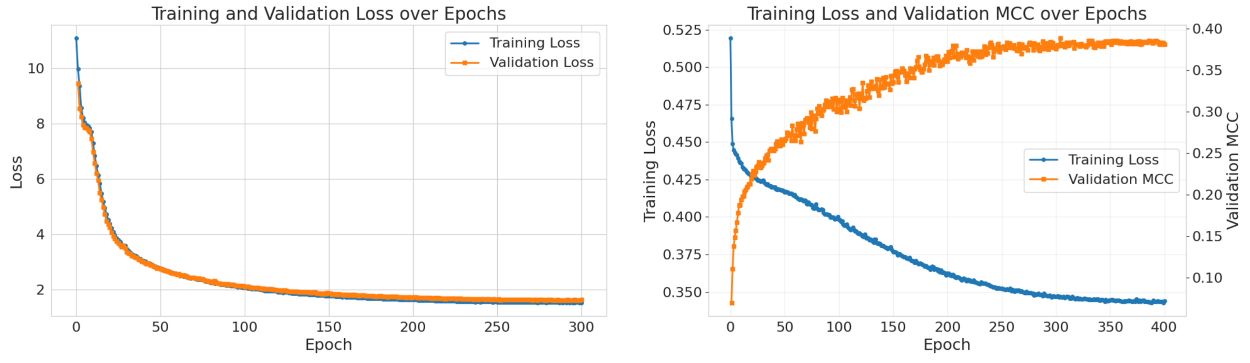

(a) (b)

Fig. S5: Training and validation curves of PEPNet under the ratio split. (a) Training and validation loss during pretraining. (b) Training loss and validation MCC during fine-tuning.

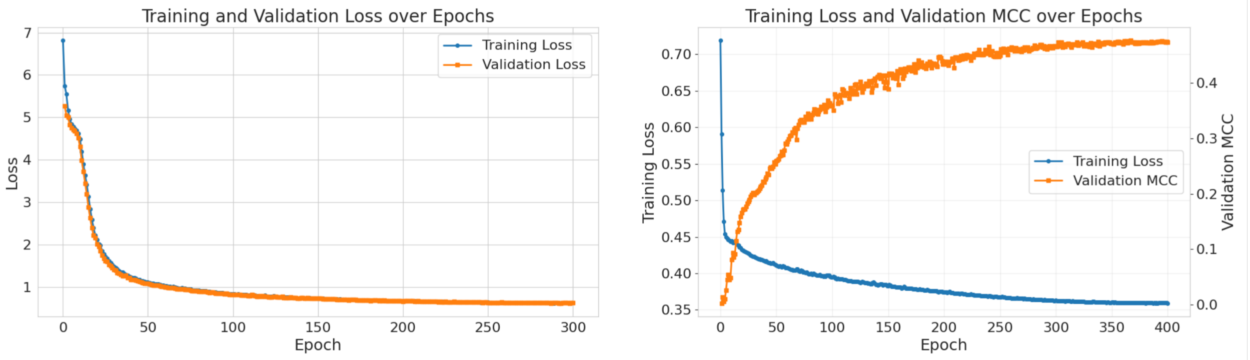

(a) (b)

Fig. S6: Training and validation curves of PEPNet+LE under the epitope-group split. (a) Training and validation loss during pretraining. (b) Training loss and validation MCC during fine-tuning.

6 Details of Experimental Results

6.1 Comparison with recent epitope prediction methods (SEMA-1D and SEMA-3D)

Antibody-agnostic epitope prediction methods aim to identify antigen residues that are likely to participate in antibody binding, without conditioning on a specific antibody. Representative ap-

proaches in this category include MaSIF-site [11], as well as more recent models such as SEMA-1D and SEMA-3D [12].

Following prior work such as AsEP, we include MaSIF-site as a baseline in the main text to ensure methodological continuity with established evaluation protocols. To further strengthen the evaluation and reflect recent advances within the same paradigm, we additionally compare our method with SEMA-1D and SEMA-3D, and report the detailed results in this Supplementary Material. SEMA-1D and SEMA-3D were evaluated using their officially released model weights (see Table S1).

Across both splitting strategies, PEPNet and SEMA models exhibit distinct and consistent performance profiles. Specifically, SEMA-1D and SEMA-3D achieve substantially higher Recall and AUC, whereas PEPNet attains markedly higher Precision. This behavior reflects a clear trade-off between sensitivity and specificity. These differences primarily arise from divergent label constructions and prediction targets, rather than architectural advantages of a particular model. SEMA-1D and SEMA-3D are antigen-only models trained with epitope labels defined as the union of epitope residues observed across multiple antibody-antigen complexes for the same antigen. This formulation encourages broader epitope predictions, naturally leading to high recall but lower precision. MaSIF-site is also an antigen-only model. Its training dataset consists of PPI pairs taken from the PRISM list of nonredundant proteins, the ZDock benchmark, PDBBind, and SabDab. However, the original publication does not explicitly state whether epitope labels for a given antigen are derived from a single antibody-antigen complex or from the union of multiple antibodies bound to the same antigen. This distinction is therefore less clearly defined than for SEMA.

In contrast, PEPNet is designed for antibody-specific epitope prediction, where epitope labels are derived from a single antigen-antibody complex. By conditioning predictions on a given antibody, PEPNet produces more conservative and specific epitope predictions, prioritizing precision over recall. As a result, while antibody-agnostic methods and antibody-specific methods can be evaluated using the same residue-level metrics, they address related but distinct biological questions, and their performance should be interpreted accordingly.

Overall, this supplementary comparison demonstrates that PEPNet complements existing antibody-agnostic epitope prediction approaches by targeting a different prediction setting, rather than serving as a direct replacement for union-epitope models such as SEMA.

Table S1: Performance under the Ratio and Group splits. Note that the highest score in each column is in bold and the second-best score is underlined.

	Ratio					Group				
	MCC	AUC	Precision	Recall	F1	MCC	AUC	Precision	Recall	F1
PEPNet	**0.401**	0.765	**0.544**	0.340	**0.419**	0.139	0.612	<u>0.230</u>	0.143	0.177
PEPNet+LE	<u>0.337</u>	0.765	<u>0.465</u>	0.295	<u>0.361</u>	0.156	0.627	**0.250**	0.155	0.191
SEMA-1D	0.235	**0.835**	0.130	**0.876**	0.226	**0.235**	**0.835**	0.132	**0.848**	<u>0.228</u>
SEMA-3D	0.223	<u>0.791</u>	0.139	<u>0.749</u>	0.235	<u>0.230</u>	<u>0.809</u>	0.137	<u>0.783</u>	**0.233**

6.2 The effectiveness of data augmentation

To examine the impact of data augmentation on improving the generalization of epitope prediction, we performed an ablation study with different augmentation settings (Table S2). In this setting, the model without augmentation (w/o aug) disables all augmentation operations described in Section Data augmentation. Similarly, the variant without augmentation except rotation (w/o aug

Table S2: Ablation results of data augmentation under ratio and group splits. Note that the highest score in each column is in bold and the second-best score is underlined.

	Ratio					Group				
	MCC	AUC	Precision	Recall	F1	MCC	AUC	Precision	Recall	F1
w/o aug	**0.487**	**0.917**	0.430	**0.645**	**0.516**	**0.322**	**0.860**	**0.381**	**0.344**	**0.362**
w/o aug(AlphaFold3)	0.111	0.609	0.152	0.204	0.174	0.063	0.576	0.127	0.107	0.116
w/o aug(w rotation)	0.390	<u>0.797</u>	<u>0.552</u>	0.317	0.403	<u>0.176</u>	<u>0.669</u>	<u>0.278</u>	<u>0.168</u>	<u>0.209</u>
w/o aug(w rotation)(AlphaFold3)	0.264	0.723	0.422	0.207	0.278	0.094	0.619	0.184	0.099	0.129
PEPNet	<u>0.401</u>	0.765	**0.544**	<u>0.340</u>	<u>0.419</u>	0.139	0.612	0.230	0.143	0.177
PEPNet(AlphaFold3)	0.346	0.751	0.497	0.286	0.363	0.119	0.601	0.208	0.125	0.156

w rotation) disables all augmentations except for independent random rotations of antigen and antibody structures.

Under the ratio split, the results reveal a clear pattern: the model without augmentation (w/o aug) achieves abnormally high performance on experimentally resolved complexes, with an MCC of 0.487 and AUC of 0.917, but suffers a dramatic drop when applied to AlphaFold3-predicted structures (MCC = 0.111, AUC = 0.609). This indicates severe overfitting to the exact geometric complementarity present in experimentally determined antigen-antibody complexes, which does not transfer to separately predicted structures. By contrast, the full PEPNet model, equipped with comprehensive data augmentation, exhibits more stable performance across both experimental and predicted inputs (MCC = 0.401 vs. 0.346; AUC = 0.765 vs. 0.751), demonstrating that augmentation improves robustness against structural noise and distribution shifts. This trend is also consistently observed under the group split.

When only rotation augmentation is retained (w/o aug w rotation), the model achieves more reasonable performance on experimentally resolved complexes (MCC = 0.390, AUC = 0.797) compared to the non-augmented version, indicating that random rotations help the model avoid overfitting to specific spatial orientations. However, its performance still drops sharply on AlphaFold3-predicted structures (MCC = 0.264, AUC = 0.723), with a larger gap than the fully augmented PEPNet (MCC = 0.346, AUC = 0.751). This suggests that while rotation alone mitigates orientation bias, the full augmentation pipeline is essential for improving cross-domain generalization.

6.3 The effectiveness of feature types

Table S3: Performance of models trained from scratch with different input features under ratio and group splits. Note that the highest score in each column is in bold.

	Ratio					Group				
	MCC	AUC	Precision	Recall	F1	MCC	AUC	Precision	Recall	F1
AF+RF	**0.362**	0.759	0.488	**0.318**	**0.385**	**0.134**	0.573	**0.227**	**0.137**	**0.171**
AF+RF(AlphaFold3)	0.339	0.747	**0.489**	0.281	0.357	0.109	0.561	0.198	0.114	0.145
AF+LE	0.313	**0.782**	0.412	0.297	0.345	0.114	0.622	0.196	0.129	0.156
AF+LE(AlphaFold3)	0.301	0.779	0.403	0.283	0.333	0.111	**0.627**	0.196	0.123	0.151

To further investigate the role of different feature types, we conducted ablation experiments by comparing traditional physicochemical features with PLM embeddings. Since our pretraining tasks involve self-supervised masking and reconstruction of physicochemical attributes, we evaluated

the two feature types using models trained from scratch without pretraining. Specifically, we designed two experimental settings: AF+RF, which incorporates atomic features (AF) and residue-level physicochemical descriptors (RF), and AF+LE, which combines atomic features with PLM embeddings (LE).

As shown in Table S3, under ratio split setting, AF+RF achieves higher performance than AF+LE in terms of classification-oriented metrics, including MCC (+0.049), Precision (+0.076), Recall (+0.021), and F1 (+0.040). In contrast, AF+LE yields superior performance in ranking-oriented evaluation, with an AUC improvement of 0.023 over AF+RF. These results suggest a complementary relationship: while traditional physicochemical features enhance the discriminative power of the model in binary classification, PLM embeddings contribute richer contextual information that benefits ranking-based evaluation. Importantly, this conclusion holds consistently under both ratio and group splits, indicating the robustness of the observation. This highlights the distinct strengths of handcrafted physicochemical features and learned PLM representations, and underscores the potential benefit of combining them in a unified framework.

7 Supplementary Tables

Table S4: Ablation results of pretraining under ratio and group splits. Note that the highest score in each column is in bold.

	Ratio					Group				
	MCC	AUC	Precision	Recall	F1	MCC	AUC	Precision	Recall	F1
w/o pretrain	0.362	0.759	0.488	0.318	0.385	0.134	0.573	0.227	0.137	0.171
w/o pretrain(AlphaFold3)	0.339	0.747	0.489	0.281	0.357	0.109	0.561	0.198	0.114	0.145
PEPNet	**0.401**	**0.765**	**0.544**	**0.340**	**0.419**	**0.139**	**0.612**	**0.230**	**0.143**	**0.177**
PEPNet(AlphaFold3)	0.346	0.751	0.497	0.286	0.363	0.119	0.601	0.208	0.125	0.156

8 Four-Stage t-SNE Visualization

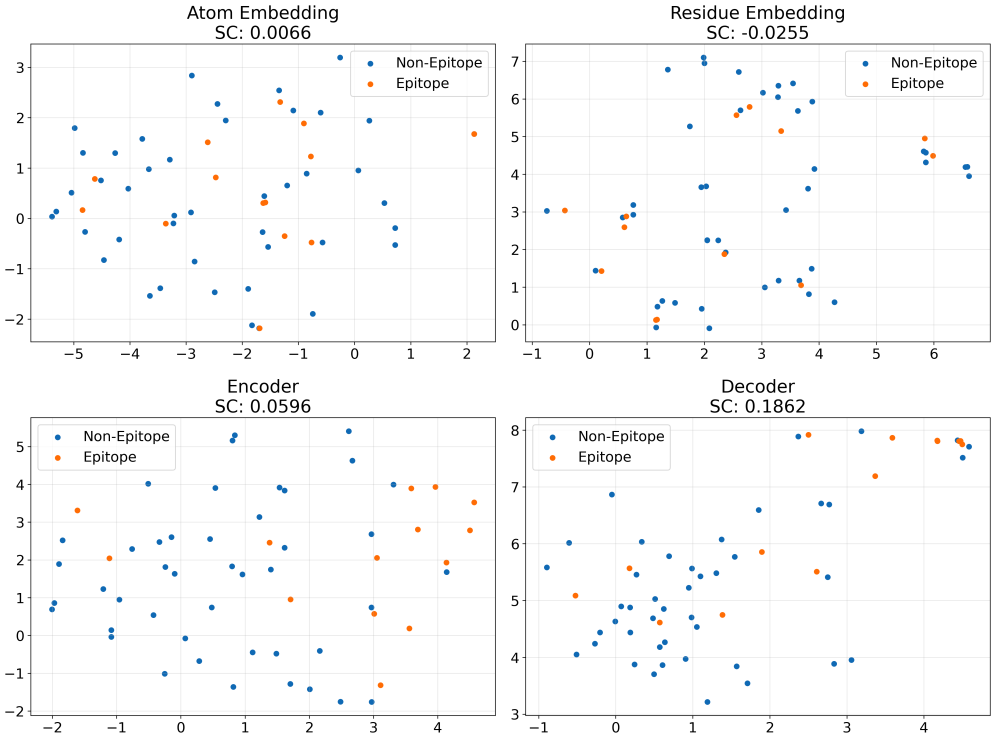

Fig. S7: The t-SNE visualizations of feature representations at different stages of the PEPNet model for 6k65_0P.

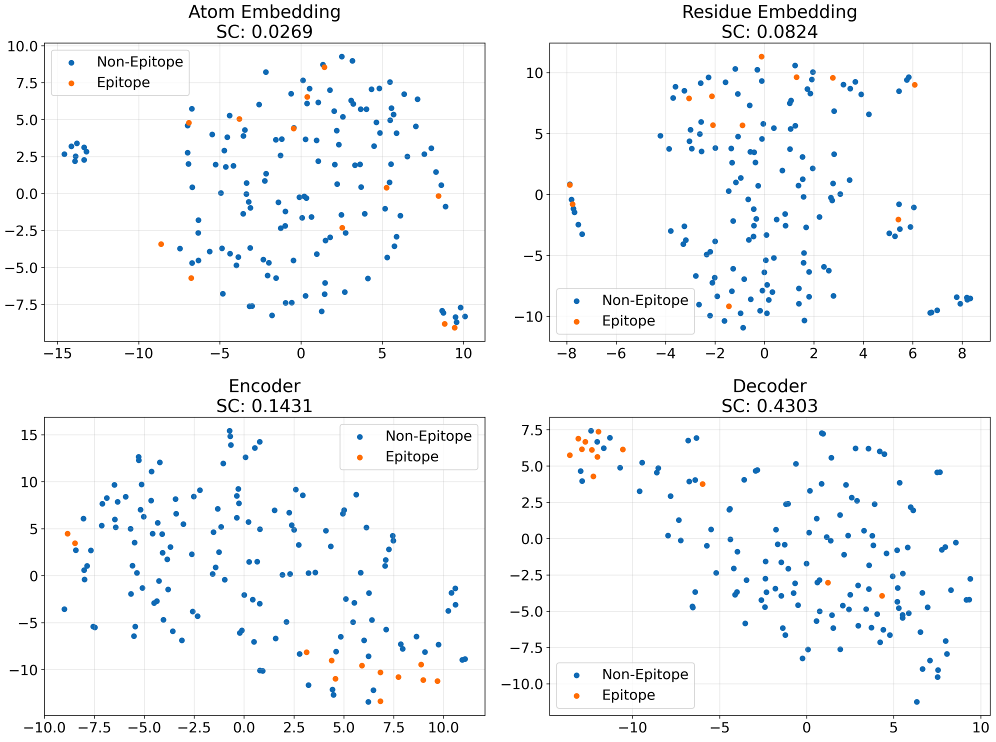

Fig. S8: The t-SNE visualizations of feature representations at different stages of the PEPNet+LE model for 4f2m_1P.

References

[1] Chunan Liu, Lilian Denzler, Yihong Chen, Andrew Martin, and Brooks Paige. AsEP: Benchmarking deep learning methods for antibody-specific epitope prediction. In A. Globerson, L. Mackey, D. Belgrave, A. Fan, U. Paquet, J. Tomczak, and C. Zhang, editors, *Advances in Neural Information Processing Systems*, volume 37, pages 11700–11734. Curran Associates, Inc., 2024.

[2] Martin Steinegger and Johannes Söding. MMseqs2 enables sensitive protein sequence searching for the analysis of massive data sets. *Nature Biotechnology*, 35(11):1026–1028, November 2017.

[3] Yang Zhang and Jeffrey Skolnick. Scoring function for automated assessment of protein structure template quality. *Proteins: Structure*, 57, 2004.

[4] Mitsunori Shiroishi, Kouhei Tsumoto, Yoshikazu Tanaka, Akiko Yokota, Takeshi Nakanishi, Hidemasa Kondo, and Izumi Kumagai. Structural Consequences of Mutations in Interfacial Tyr Residues of a Protein Antigen-Antibody Complex: THE CASE OF HyHEL-10-HEL*. *Journal of Biological Chemistry*, 282(9):6783–6791, March 2007.

[5] Eric J. Sundberg, Mariela Urrutia, Bradford C. Braden, Jordi Isern, Daisuke Tsuchiya, Barry A. Fields, Emilio L. Malchiodi, José Tormo, Frederick P. Schwarz, and Roy A. Mariuzza. Estimation of the Hydrophobic Effect in an Antigen-Antibody Protein-Protein Interface,. *Biochemistry*, 39(50):15375–15387, December 2000.

[6] Michel F. Sanner, Arthur J. Olson, and Jean-Claude Spehner. Reduced surface: An efficient way to compute molecular surfaces. *Biopolymers*, 38(3):305–320, 1996.

[7] Stephen F. Altschul, Thomas L. Madden, Alejandro A. Schäffer, Jinghui Zhang, Zheng Zhang, Webb Miller, and David J. Lipman. Gapped BLAST and PSI-BLAST: A new generation of protein database search programs. *Nucleic Acids Research*, 25(17):3389–3402, September 1997.

[8] Zeming Lin, Halil Akin, Roshan Rao, Brian Hie, Zhongkai Zhu, Wenting Lu, Nikita Smetanin, Robert Verkuil, Ori Kabeli, Yaniv Shmueli, Allan dos Santos Costa, Maryam Fazel-Zarandi, Tom Sercu, Salvatore Candido, and Alexander Rives. Evolutionary-scale prediction of atomic-level protein structure with a language model. *Science*, 379(6637):1123–1130, 2023.

[9] Jacob Devlin, Ming-Wei Chang, Kenton Lee, and Kristina Toutanova. BERT: Pre-training of Deep Bidirectional Transformers for Language Understanding, May 2019.

[10] Jeffrey A. Ruffolo, Jeffrey J. Gray, and Jeremias Sulam. Deciphering antibody affinity maturation with language models and weakly supervised learning, 2021.

[11] P. Gainza, F. Sverrisson, F. Monti, E. Rodolà, D. Boscaini, M. M. Bronstein, and B. E. Correia. Deciphering interaction fingerprints from protein molecular surfaces using geometric deep learning. *Nature Methods*, 17(2):184–192, February 2020.

[12] Nikita V Ivanisenko, Tatiana I Shashkova, Andrey Shevtsov, Maria Sindeeva, Dmitriy Umerenkov, and Olga Kardymon. SEMA 2.0: Web-platform for B-cell conformational epitopes prediction using artificial intelligence. *Nucleic Acids Research*, 52(W1):W533–W539, July 2024.
